# Promotion of Cellular and Humoral Immunity against Foot-and-Mouth Disease Virus by Immunization with Virus-Like Particles Encapsulated in Monophosphoryl Lipid A and Liposomes

**DOI:** 10.3390/vaccines8040633

**Published:** 2020-10-31

**Authors:** Woo Sik Kim, Yong Zhi, Huichen Guo, Eui-Baek Byun, Jae Hyang Lim, Ho Seong Seo

**Affiliations:** 1 Research Division for Radiation Science, Korea Atomic Energy Research Institute, Jeongeup 56212, Korea; yongzhi@kaeri.re.kr (Y.Z.); ebbyun80@kaeri.re.kr (E.-B.B.); 2 Department of Radiation Science, University of Science and Technology, Daejeon 34057, Korea; 3 State Key Laboratory of Veterinary Etiological Biology, National Foot and Mouth Disease Reference Laboratory, Lanzhou Veterinary Research Institute, Chinese Academy of Agricultural Sciences, Lanzhou 730000, China; guohuichen@caas.cn; 4 Department of Microbiology, Ewha Womans University College of Medicine, Seoul 03760, Korea; jlim19@ewha.ac.kr; 5 Ewha Education & Research Center for Infection, Ewha Womans University Medical Center, Seoul 03760, Korea

**Keywords:** foot-and-mouth disease, virus-like particles, vaccine, liposome, TLR4 agonist, immunogenicity

## Abstract

Virus-like particles (VLPs) have emerged as promising vaccine candidates against foot-and-mouth disease (FMD). However, such vaccines provide a relatively low level of protection against FMD virus (FMDV) because of their poor immunogenicity. Therefore, it is necessary to design effective vaccine strategies that induce more potent immunogenicity. In order to investigate the means to improve FMD VLP vaccine (VLP_FMDV_) immunogenicity, we encapsulated VLPs (MPL/DDA-VLP_FMDV_) with cationic liposomes based on dimethyldioctadecylammonium bromide (DDA) and/or monophosphoryl lipid A (MPL, TLR4 agonist) as adjuvants. Unlike inactivated whole-cell vaccines, VLP_FMDV_ were successfully encapsulated in this MPL/DDA system. We found that MPL/DDA-VLP_FMDV_ could induce strong cell-mediated immune responses by inducing not only VLP-specific IFN-γ^+^CD4^+^ (Th1), IL-17A^+^CD4^+^ (Th17), and IFN-γ^+^CD8^+^ (activated CD8 response) T cells, but also the development of VLP-specific multifunctional CD4^+^ and CD8^+^ memory T cells co-expressing IFN-γ, TNF-α, and IL-2. In addition, the MPL/DDA-VLP_FMDV_ vaccine markedly induced VLP-specific antibody titers; in particular, the vaccine induced greater Th1-predominant IgG responses than VLP_FMDV_ only and DDA-VLP_FMDV_. These results are expected to provide important clues for the development of an effective VLP_FMDV_ that can induce cellular and humoral immune responses, and address the limitations seen in current VLP vaccines for various diseases.

## 1. Introduction

Foot-and-mouth disease virus (FMDV) can cause highly contagious foot-and-mouth disease (FMD) in cloven-hoofed livestock, particularly cattle, sheep, goats and pigs [1,2]. This virus belongs to a prototypical member of the Aphthovirus of the Picornaviridae family, and is classified into seven distinct serotypes (O, A, C, SAT 1 to 3, and Asia 1), as well as >65 subtypes [3,4]. Given that cross-reactivity varies, antigenic diversity among these serotypes and subtypes should be considered for vaccine development [5]. The FMDV virion surrounds an approximately 8.3 kb positive-sense single-stranded RNA genome within an envelope composed of icosahedral capsids from 60 copies of four distinct structural polypeptides, from VP1 to VP4, organized in 12 pentameric forms [3,6,7].

Although chemically inactivated FMDV vaccines have proven to be effective in controlling FMD in endemic areas, the disease still affects millions of animals and continues to cause economic problems worldwide [8,9]. Moreover, these commercial vaccines have several immunological limitations, such as absent or very low immune memory and cell-mediated immunity, and limited cross-reactive immunity against multiple serotypes [10,11,12]. Thus, there is currently a clear demand for a new generation or platform of FMD vaccines, such as recombinant subunit vaccines, DNA vaccines, adenovirus vector vaccines, and virus-like particles (VLPs) that can replace or improve current FMD vaccines [7,13,14].

Among the various vaccine platforms, FMD VLPs (VLP_FMDV_), which retain the antigenicity of capsid proteins of the native FMDV virion, but lack a viral genome, have been evaluated as being among the most innovative vaccine platforms [15,16]. The VLP_FMDV_ vaccine induces a strong and sustained humoral immune response, but is not sufficient to provide 100% protective immunity in the setting of a large animal infection model [12,17].

Since T-helper 1 (Th1)-type T cell immunity and Th1-based IgG subclasses are known to be important in regulating RNA viral infections, including FMDV, a number of studies are underway concerning immunogenic adjuvant systems to improve cellular and humoral immune responses to VLP_FMDV_ vaccines [12,18]. Terhuja et al. reported that a VLP_FMDV_ vaccine formulated with CpG adjuvants increased T-lymphocyte proliferation and IgG2 response, thereby conferring superior protection (75% protection) compared to VLP_FMDV_-only vaccination in guinea pigs [12,18]. Moreover, Guo et al. reported that immunization with VLP_FMDV_ emulsified in mineral oil adjuvant (Complete Freund’s Adjuvant) elicited similar levels of protective immunity to those of inactivated whole-virus vaccines, and this protection correlated with increased levels of FMDV-specific antibodies, T cell proliferation, and Th1-type cytokine (IFN-γ) production [13]. Given these findings, the adjuvant system is an essential component in optimizing and maximizing the protective efficacy of VLP_FMDV_ vaccines.

AS01 is a liposome-based adjuvant system developed by GlaxoSmithKline Inc. (GSK) containing a synthetic TLR4 agonist (monophosphoryl lipid A; MPL) and Quillaja saponaria Molina, fraction 21 (QS-21) [19]. It has been tested as a potent cell-mediated immune response adjuvant for clinical human vaccine studies, including malaria, tuberculosis and herpes zoster [19,20]. The first licensed AS01-based vaccine, Shingrix, displayed >90% protection against herpes zoster virus infection at all ages [21]. In addition, hybrid malaria–hepatitis B VLPs formulated with AS01 adjuvant represent the first malaria vaccine in a phase III clinical trial [22]. However, the use of AS01 as a veterinary vaccine is very limited, because the cost of this system is extremely high and the patent for QS-21 is owned by GSK [20,23,24].

In this study, a VLP_FMDV_ vaccine was manufactured by applying a different type of AS01 adjuvant system that uses dimethyldioctadecylammonium bromide (DDA) instead of QS-21 to avoid patent and pricing issues. Furthermore, we investigated whether the incorporation of MPL into the DDA liposome-VLP complex, named MPL/DDA-VLP_FMDV_, could enhance VLP-specific humoral and cellular immunity in mice.

## 2. Materials and Methods

### 2.1. VLP Purification

FMDV capsid proteins (His-Sumo-VP0, His-Sumo-VP1, and His-Sumo-VP3) were obtained using an *Escherichia coli* (*E. coli*) expression system established at Lanzhou Veterinary Research Institute (LVRI, Lanzhou, China), and the assembly of VLPs was performed according to previously described methods [13]. The assembled VLPs were identified using a Zetasizer-Nano instrument (DLS; Malvern Zetasizer-Nano ZS90, Worcestershire, UK) and by transmission electron microscopy (TEM) (HT7700, Tokyo, Japan), respectively.

### 2.2. Preparation of Vaccine Formulations and Immunization of Mice

C57BL/6 mice (female, 6–7 weeks of age) were purchased from Orient Bio Inc. (Seoul, Korea). Mice were acclimatized to the following controlled conditions: temperature (25 ± 2 °C), humidity (55 ± 5%), and 12 h light/dark cycle at the Central Animal Research Laboratory of the Korea Atomic Energy Research Institute (KAERI, Jeongeup, Korea). The animal experiments were approved by the Institutional Animal Care and Use Committee (KAERI-IACUC-2019-018) and were performed in strict compliance with the guidelines prescribed by the committee. For adjuvant formulation, MPL and DDA were obtained from Sigma-Aldrich (St. Louis, MO, USA). DDA can be applied to the preparation of cationic liposomes, as described previously [25]. Briefly, DDA (10 mg/mL) mixed with distilled water (DW) was heated at 80 °C until micelles formed, and then cooled to room temperature (RT). MPL mixed with DW containing 0.2% trimethylamine was heated at 70 °C for 30 seconds, then sonicated for 30 seconds, and these steps were repeated twice. MPL was subsequently mixed with DDA immediately before use (termed MPL/DDA). The size distribution of the formulated VLP vaccines was confirmed by Dynamic Light Scattering (DLS) analysis. Mice were immunized subcutaneously twice at 2-week intervals with 100 μL of PBS (Group 1/G1), 250 μg DDA (G2), 25/250 μg MPL/DDA (G3), 10 μg VLP_FMDV_ (G4), 250/10 μg DDA/VLP_FMDV_ (G5; DDA-VLP_FMDV_) or 25/250/10 μg MPL/DDA/VLP_FMDV_ (G6; MPL/DDA-VLP_FMDV_).

### 2.3. Flow Cytometry

Two weeks after the final immunization, the spleens of immunized mice were filtered through a cell strainer (40 µm, BD Biosciences, San Diego, CA, USA) in RPMI 1640 (GIBCO, Carlsbad, CA, USA) complete medium (2% fetal bovine serum, GIBCO). Red blood cells (RBCs) present in the filtrate were lysed for 3 min with RBC lysis buffer (Sigma Aldrich) and washed with RPMI 1640 complete medium. Single-cell suspensions (2 × 10^6^ cells) were stimulated with 10 μg/mL VLPs and 2 μg/mL anti-CD28 monoclonal antibody (eBioscience, San Diego, CA, USA) in a buffer containing 0.5 μg/mL GolgiStop (eBioscience) and 0.5 μg/mL GolgiPlug (BD Biosciences) for 12 h at 37 °C. Next, the cells were washed in PBS and stained for 30 min at RT with a Live/Dead staining kit (InvivoGen, San Diego, CA, USA), anti-CD3e-APC-Cy7 (eBioscience), anti-CD4-Alexa488 (eBioscience), anti-CD8-PerCp-Cy5.5 (eBioscience) and anti-CD44-V450 (BD Bioscience) antibodies to stain T cell surface markers. The cells were fixed and permeabilized using a Cytofix/Cytoperm kit (BD Bioscience) according to the manufacturer’s recommendations, and intracellular cytokines were stained with anti-IFN-γ-PE (BD Bioscience), TNF-α-APC (BD Bioscience), anti-IL-2-PE-Cy7 (eBioscience), anti-IL-5 (BD Bioscience) and anti-IL-17A-PE-Cy7 (eBioscience) Abs. Subsequently, the stained cells were analyzed using a BD FACSVerse flow cytometer and FlowJo analysis software (TreeStar, Ashland, OR, USA) to determine T cell immunity induced by various vaccine formulations.

### 2.4. Cytokine ELISA

Splenocyte culture supernatants, prepared as described above, were collected, and cytokine levels (IFN-γ, IL-5, and IL-17A) were measured using an enzyme-linked immunosorbent assay (ELISA) kit (eBioscience).

### 2.5. VLP-Specific IgG Isotype ELISA

The VLP-specific IgG, IgM, IgG1 and IgG2a responses in the serum from antigen-immunized mice were analyzed by sandwich ELISA. Ninety-six-well plates were coated with 1 μg/mL VLP at 4 °C for 24 h. The plates were washed 5 times with PBS containing 0.2% Tween 20 (PBS-T), and then blocked with 5% BSA in PBS at RT for 1 h. After blocking, diluted serum was added to each well and incubated at RT for 1 h. Unbound antibodies were removed by washing with PBS-T, and then goat anti-mouse IgG-HRP (Sigma-Aldrich), goat anti-mouse IgM-HRP (Sigma-Aldrich), goat anti-mouse IgG1-HRP (Sigma-Aldrich), or IgG2a-HRP (Sigma-Aldrich) were added to the wells, and incubated for 30 min at RT. After washing the plates 5 times with PBS-T, 100 μL of TMB substrate reagent (BD Biosciences, Franklin Lakes, NJ, USA) was added. When colors developed, 50 μL of 2 N H_2_SO_4_ was added, and optical absorbance was measured at 450 nm using a Victor X3 light plate reader (Perkin-Elmer, Waltham, MA, USA).

### 2.6. Statistical Analysis

All analyses were repeated at least twice. The level of significance for comparisons between samples was determined by unpaired Student’s *t*-tests (between two groups), and one-way ANOVA followed by Dunnett’s multiple comparison test (between three or more groups) using statistical software (GraphPad Prism, version 5; San Diego, CA, USA). Results are expressed as means ± standard deviation (S.D.). Values of * *p* < 0.05, ** *p* < 0.01 and *** *p* < 0.001 were considered to be statistically significant.

## 3. Results

### 3.1. Higher VLP-Specific T Cell Immunity Induced by Formulating in MPL/DDA

**Figure 1:**
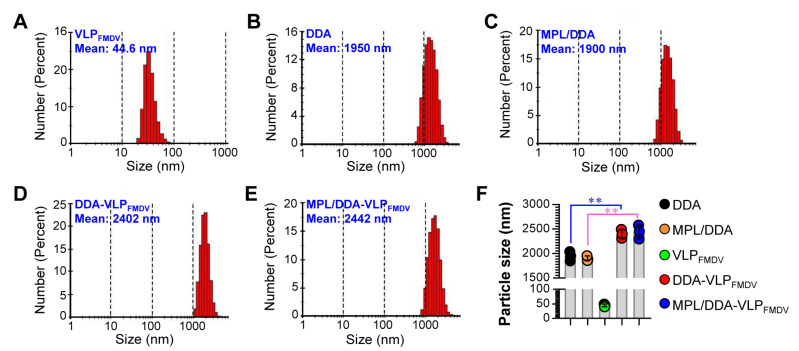
Physical characterization of virus-like particle (VLP) vaccines formulated with dimethyldioctadecylammonium bromide (DDA) or/and monophosphoryl lipid A (MPL). Particle size distributions of VLP_FMDV_ (**A**), DDA (**B**), MPL/DDA (**C**), DDA-VLP_FMDV_ (**D**), and MPL/DDA-VLP_FMDV_ (**E**) were measured by DLS analysis. VLP_FMDV_: recombinant food-and-mouth disease VLP vaccine; MPL/DDA: MPL and DDA formulation; DDA-VLP_FMDV_: VLP_FMDV_ formulated with DDA; MPL/DDA-VLP_FMDV_: VLP_FMDV_ formulated with DDA and MPL. (**F**) Bar graph is expressed as the mean ± SD of 3 samples per group. ** *p* < 0.01.

We first performed a DLS analysis on five types (DDA only; MPL/DDA: MPL and DDA formulation; VLP_FMDV_ only; DDA/VLP_FMDV_: VLP_FMDV_ formulated with DDA; MPL/DDA/VLP_FMDV_: VLP_FMDV_: VLP_FMDV_ formulated with DDA and MPL) of formulated VLP_FMDV_ vaccines to confirm the particle sizes and successful formulation. As shown in Figure 1A, VLP_FMDV_ had an average diameter of 44.6 nm. In addition, DDA (Figure 1B) and MPL/DDA (Figure 1C) without VLP_FMDV_ showed average diameters of 1950 nm and 1900 nm, respectively. After formulating with VLP, the average diameters of DDA-VLP_FMDV_ (Figure 1D,F) and MPL/DDA/VLP_FMDV_ (Figure 1E,F) increased significantly to 2402 nm and 2442 nm, respectively. There was no significant effect on particle size between DDA-VLP_FMDV_ and MPL/DDA-VLP_FMDV_ (Figure 1F). These results strongly suggested that VLPs were successfully encapsulated into DDA or MPL/DDA liposomes, which led to the increase in liposome particle size.

**Figure 2:**
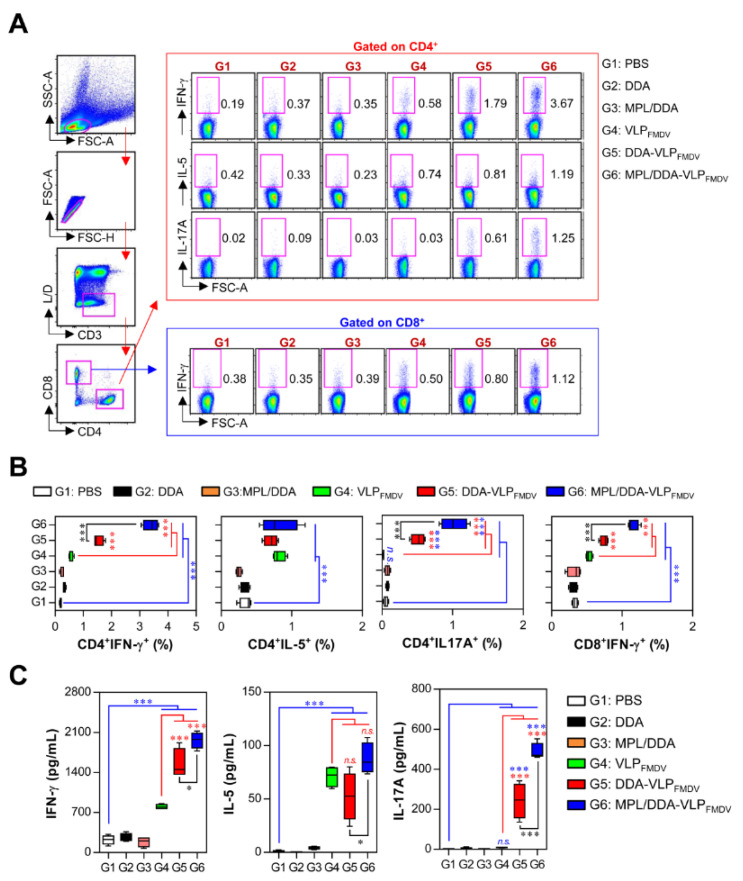
Analysis of VLP-specific CD4^+^ and CD8^+^ T cell responses. Mice (*n* = 5 animals/group) were immunized twice with PBS (G1), DDA (G2), MPL/DDA (G3), VLP_FMDV_ (G4), DDA-VLP_FMDV_ (G5), or MPL/DDA/VLP_FMDV_ (G6). (**A**,**B**) Single-cell suspensions were re-stimulated with 10 μg/mL VLP for 12 h and analyzed for VLP-specific Th1 (IFN-γ-producing CD4^+^ T cells), Th2 (IL-5-producing CD4^+^ T cells), Th17 (IL-17A-producing CD4^+^ T cells) and activated CD8^+^ T cells (IFN-γ-producing CD8^+^ T cells) by intracellular cytokine (IFN-γ, IL-5, IL-17A) staining based on T cell-specific makers (CD3, CD4, CD8). (**B**) The percentages of VLP-specific Th1, Th2 and Th17, and activated CD8^+^ T cells in spleens from immunized mice were analyzed by flow cytometry. (**C**) Single-cell suspensions of splenocytes were treated with 10 μg/mL VLP for 24 h, and supernatants were collected and evaluated for VLP-specific cytokines (IFN-γ, IL-5 and IL-17A) by ELISA. The means ± SD shown are representative of two independent experiments. * *p* < 0.05, *** *p* < 0.001. n.s.: not significant.

Next, since both CD4^+^ and CD8^+^ T cells are crucial to protect against viral infection, including FMDV [26,27], we evaluated T cell subtypes induced by VLP_FMDV_ vaccination formulated with DDA and MPL. Mice were immunized subcutaneously twice at 2-week intervals, and single cell suspensions of splenocytes were re-stimulated with 10 µg VLP, followed by analyzing VLP-specific Th1 (IFN-γ-producing CD4^+^ T cells), Th2 (IL-5-producing CD4^+^ T cells), Th17 (IL-17A-producing CD4^+^ T cells), and activated CD8^+^ T cells (IFN-γ-producing CD8^+^ T cells) by cytometry gating, as shown in Figure 2A. As seen in Figure 2B, all groups immunized with VLP_FMDV_ (VLP_FMDV_ only (G4), DDA-VLP_FMDV_ (G5), and MPL/DDA-VLP_FMDV_ (G6)) were found to have significantly increased frequencies of IFN-γ^+^CD4^+^, IL-5^+^CD4^+^, and IFN-γ^+^CD8^+^ cells compared to the PBS control group (G1). Among these groups, the MPL/DDA-VLP_FMDV_ (G6)-immunized group had the highest frequency of IFN-γ^+^CD4^+^ Th1 cells (G4 vs. G6; up to 2.8-fold, G5 vs. G6; up to 2-fold) and IL-17A^+^CD4^+^ cells (G4 vs. G6; up to 30-fold, G5 vs. G6; up to 2-fold) compared to the other groups (G4 and G5). Nevertheless, we did not identify any significant differences in IL-5^+^CD4^+^ Th2 cells between VLP_FMDV_-vaccinated groups (G4, G5, G6), indicating that the MPL/DDA formulation led to a Th1-biased immune response to VLP_FMDV_. In addition, improved frequencies of IFN-γ^+^CD8^+^ T cells (G4 vs. G6; up to 1.6-fold, G5 vs. G6; up to 1.5-fold) were found in the MPL/DDA-VLP_FMDV_ group (G6) compared to the VLP_FMDV_ alone (G4) and DDA-VLP_FMDV_ (G5) groups (Figure 2B). However, significant differences between all T cell subsets (Th1, Th2, and Th17) in unstimulated T cells were not observed (data not shown).

The levels of secreted cytokines in splenocytes isolated from each group responding to VLP stimulation were measured by ELISA (Figure 2C). Consistent with the above results, significantly higher levels of IFN-γ and IL-17A were detected in the MPL/DDA-VLP_FMDV_ groups (G6) than in the other groups (G4 and G5), while the IL-5 production was similar to or slightly higher than that of the VLP_FMDV_ alone (G4) and DDA-VLP_FMDV_ (G5) groups. This confirmed that the MPL/DDA formulation can induce VLP_FMDV_ immune responses in the direction of Th1 and Th17, but less so for Th2.

### 3.2. Generation of VLP-Specific Multifunctional CD4^+^ T Cells by MPL/DDA-VLP_FMDV_

**Figure 3:**
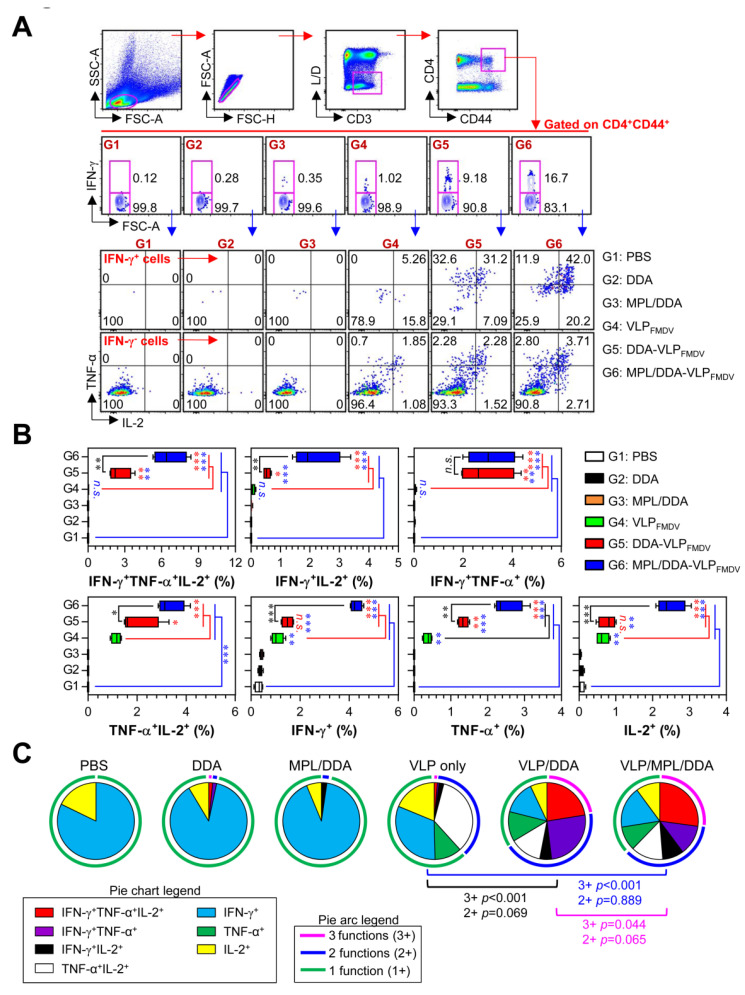
Analysis of antigen-specific multifunctional CD4^+^ T cells. (**A**) Gating strategy for multifunctional CD4^+^ memory T cells. Memory CD4+ T cells (determined by increased CD44 expression) gated to show CD3^+^CD4^+^CD44^+^ T cells. For multifunctional CD4^+^ T cells analysis, cells were identified by intracellular cytokine (IFN-γ, TNF-α, and IL-2) staining based on memory CD4^+^ T cell gating. (**B**) The percentage of VLP-specific total cytokine (IFN-γ, TNF-α, and/or IL-2)-producing cells among splenic CD4^+^CD44^+^ memory T cells. (**C**) Pie charts representing the mean frequencies of cells co-expressing IFN-γ, TNF-α, and/or IL-2. The relative amounts of single-, double- and triple-functional CD4^+^CD44^+^ memory T cells are indicated as pie arcs. Means ± SD (*n* = 5 mice/group) shown are representative of two independent experiments. * *p* < 0.05, ** *p* < 0.01, *** *p* < 0.001. n.s.: not significant.

We next investigated whether the MPL/DDA-VLP_FMDV_ vaccine induces multifunctional CD4^+^ T cells capable of simultaneously producing multiple effector Th1 cytokines (IFN-γ, TNF-α, and IL-2), which are known to have higher effector functions than monofunctional CD4^+^ T cells [28]. Single cell suspensions of splenocytes obtained from immunized mice were re-stimulated with 10 µg VLP, and then VLP-specific single-, double- or triple-positive CD3^+^CD44^+^CD4^+^ T cells were analyzed by multicolor intracellular cytokine staining and flow cytometry, as described in Figure 3A. CD3 and CD44 antibodies were used as markers to identify total T cells and memory T cells, respectively. As shown in Figure 3B, IFN-γ^+^IL-2^+^ (G4 vs. G6; up to 56-fold, G5 vs. G6; up to 4-fold), TNF-α^+^IL-2^+^ double-positive (G4 vs. G6; up to 6.9-fold, G5 vs. G6; up to 1.4-fold), and triple-positive CD44^+^CD4^+^ T cells (G4 vs. G6; up to 64-fold, G5 vs. G6; up to 2.7-fold) were significantly enhanced in the MPL/DDA-VLP_FMDV_-vaccinated group (G6) compared to those in the G4 and G5 groups. However, there was no difference between G5 and G6 in the IFN-γ^+^TNF-α^+^ double-positive CD44^+^CD4^+^ T cells. The proportions of single cytokines, IFN-γ, TNF-α or IL-2-positive CD44^+^CD4^+^ T cells were significantly increased in the VLP_FMDV_-immunized groups (G4, G5, G6), but the MPL/DDA-VLP_FMDV_ vaccine (G6) was the highest among all the groups. Based on the above data, we analyzed the relative proportions of single-, double- and triple-positive cytokine levels in total CD44^+^CD4^+^ T cells (Figure 3C). The main CD44^+^CD4^+^ T cells in the negative control groups (G1, G2, and G3) were single-positive, cytokine-producing T cells. However, when mice were vaccinated with MPL/DDA-VLP_FMDV_ (G6), the triple-positive cytokine CD44^+^CD4^+^ T cells dramatically increased even compared to the G4 (*p* < 0.001) and G5 (*p* < 0.044) groups. These data demonstrated that the DDA formulation also effectively induced VLP-specific cellular immunity, whereas the MPL/DDA formulation was more effective in generating VLP-specific multifunctional CD4^+^ T cell immunity.

### 3.3. Generation of VLP-Specific Multifunctional CD8^+^ T Cells by MPL/DDA-VLP_FMDV_

**Figure 4:**
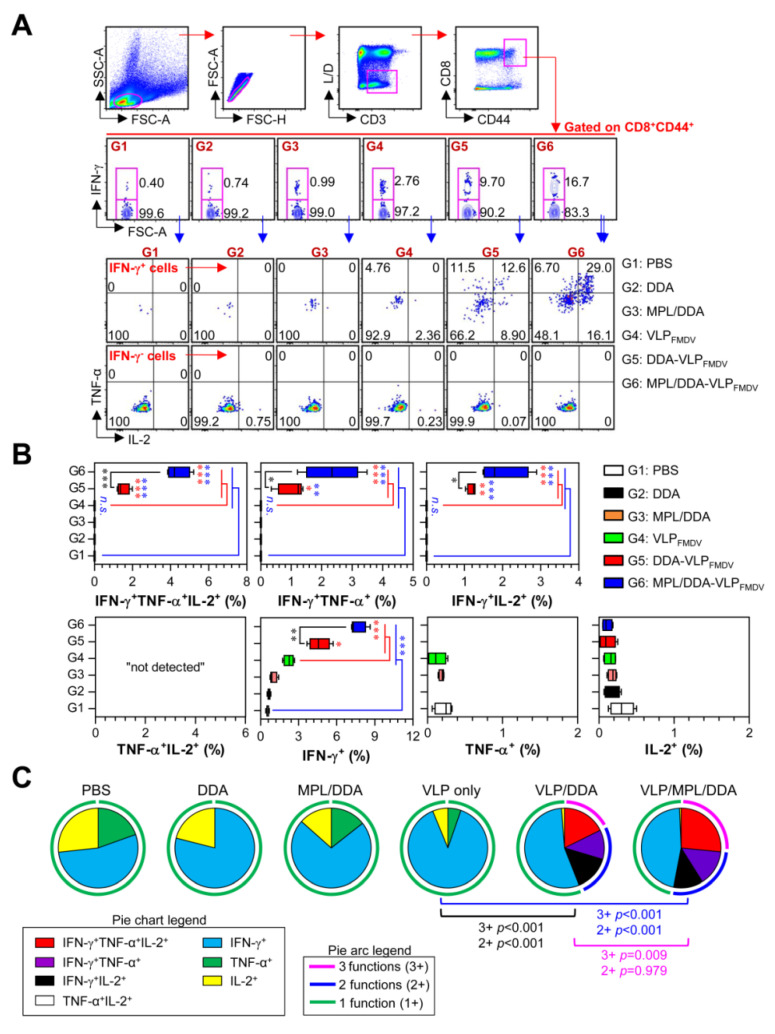
Analysis of antigen-specific multifunctional CD8^+^ T cells. (**A**) Gating strategy for multifunctional CD8^+^ memory T cells. (**B**) Percentage of VLP-specific total cytokine (IFN-γ, TNF-α, and/or IL-2)-producing cells among splenic CD8^+^CD44^+^ memory T cells. (**C**) Pie charts represent the mean frequencies of cells co-expressing IFN-γ, TNF-α, and/or IL-2. The relative amounts of single-, double- and triple-functional CD4^+^CD44^+^ memory T cells are indicated as pie arcs. The means ± SD (*n* = 5 mice/group) shown are representative of two independent experiments. * *p* < 0.05, ** *p* < 0.01, *** *p* < 0.001. n.s.: not significant.

We next analyzed the functional composition of VLP-specific multifunctional CD8^+^ T cells. Multifunctional viral-specific CD8^+^ T cells have been shown to be important and effective immune cells during viral infection [29,30]. Unlike single-positive CD4^+^ T cells, there was no increase in TNF-α or IL-2 single-positive CD8^+^ cells, and IFN-γ single-positive CD8^+^ cells were only increased in VLP_FMDV_-vaccinated groups (G4, G5, G6). Among these, the MPL/DDA-VLP_FMDV_ group (G6) had the highest proportion of VLP-specific IFN-γ single-positive CD8^+^ T cells compared to the other groups (G1–G5). Surprisingly, the VLP_FMDV_ only group (G4) showed no or few double and triple multifunctional CD8^+^ T cells, while the G5 and G6 groups had significantly higher levels of those cells than the G4 group. In addition, we found that MPL/DDA-VLP_FMDV_ (G6) represented the highest frequency of IFN-γ^+^TNF-α^+^ (G4 vs. G6; up to 86-fold, G5 vs. G6; up to 2.26-fold), IFN-γ^+^IL-2^+^ double-positive (G4 vs. G6; up to 75-fold, G5 vs. G6; up to 1.8-fold), and triple-positive CD44^+^CD8^+^ T cells (G4 vs. G6; up to 115.6-fold, G5 vs. G6; up to 2.8-fold) compared to the other groups (Figure 4A,B). Based on the above data, we analyzed the relative proportions of single-, double- and triple-positive CD44^+^CD8^+^ T cells (Figure 4C). We found only single-positive multifunctional CD8^+^ cells in the G1, G2, G3 and G4 groups. The proportion of double- and triple-positive CD44^+^CD8^+^ T cells dramatically increased in the G5 and G6 groups. Together, these data show that the addition of MPL and DDA adjuvants to the VLP_FMDV_ vaccine can effectively induce superior multifunctional T cell responses.

### 3.4. VLP-Specific Antibody Responses Elicited by Immunization with MPL/DDA-VLP_FMDV_

**Figure 5:**
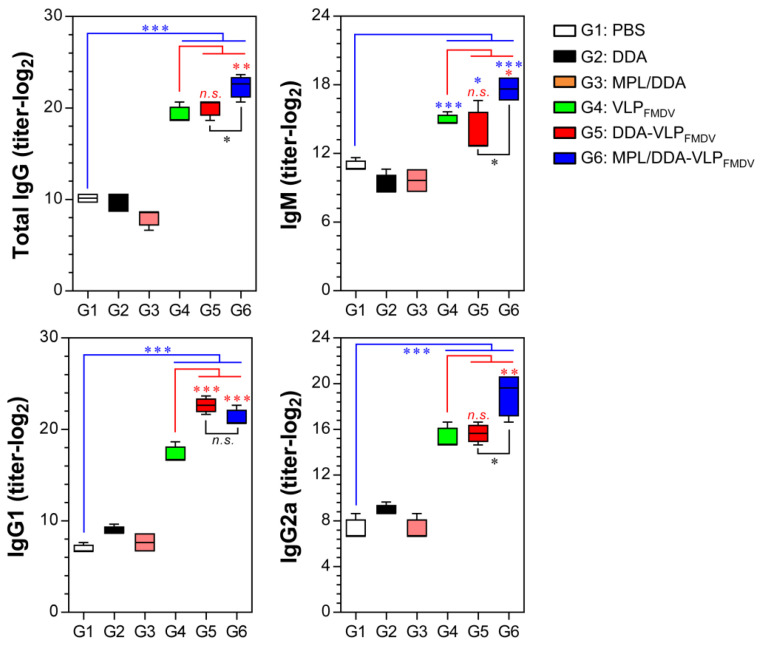
Analysis of VLP-specific serum antibody isotype. Mice were immunized twice with VLP_FMDV_ in different adjuvant combinations. Two weeks after final immunization, serum from five mice in each group was obtained, and the VLP-specific IgG, IgM, IgG1 and IgG2a were analyzed using ELISA. Data from one of two independent experiments are shown. * *p* < 0.05, ** *p* < 0.01, *** *p* < 0.001. n.s.: not significant.

Th1-predominant IgG isotypes, such as IgG2a, IgG2b, IgG2c and IgM, are more likely to induce stronger protective effects against viral infection, including FMDV, than Th2-predominant IgG responses [12,31]. Thus, we evaluated whether the formulations of DDA and/or MPL could promote the generation of humoral Th1- and/or Th2-predominant immune responses. As shown in Figure 5, the MPL/DDA-VLP_FMDV_ group (G6) had significantly higher levels of total IgG, IgM, and Th1-predominant IgG2a than the G4 and G5 groups. In contrast, the Th2-predominant IgG1 level of G6 was similar to that of G5 (Figure 5). These findings suggest that the MPL and DDA formulations induced greater VLP-specific Th1-predominant IgG responses.

## 4. Discussion

FMD is one of the most economically devastating veterinary diseases worldwide [32]. Although a chemically inactivated whole FMD vaccine has been widely used and is known to provide a reduction in FMD prevalence in endemic areas, there is a clear need for a new generation of FMD vaccines to improve overall immunogenicity and safety [33]. Among the several types of vaccines employed for veterinary and human needs, VLP_FMDV_ vaccines have been proven to show reliable immunogenicity and safety in pre-clinical and clinical studies [34,35,36]. Since VLPs can be manufactured in biosafety level 1 (BSL1) facilities, they are also certainly more safe and economical than the current inactivated FMD vaccine [37]. Previous studies have shown that VLP_FMDV_ can be stably expressed and packaged using an *E. coli* system, but this has not yet been commercialized because of the lack of exceptionally high immunogenicity compared to current vaccines [12,17]. To improve vaccine immunogenicity, several adjuvant systems have been formulated for use with VLP_FMDV_ [12]. In order to enhance the cellular immunity of the VLP_FMDV_ vaccine, we used DDA, a liposome-type adjuvant, to encapsulate VLP_FMDV_. In addition, MPL as an adjuvant was combined with DDA liposomes to increase VLP-specific multifunctional T cell responses.

Liposomes are an attractive delivery system that has been used to induce the effective uptake of various vaccine candidates, such as proteins, peptides, and genes (DNA and RNA), for presentation to APCs such as dendritic cells and macrophages [38,39,40]. Furthermore, recently, researchers have investigated the incorporation of synthetic TLR agonists, such as MPL and CpG oligodeoxynucleotides, into the membrane bilayer of liposomes to enhance their adjuvant effect [41,42,43]. MPL, a detoxified bacterial lipopolysaccharides, has been shown to have beneficial effects in a range of vaccines, including for allergies, cancer and pathogens [44,45,46]. For example, in a set of studies evaluating the impact of adjuvants on vaccine immunogenicity, *Mycobacterium tuberculosis* (Mtb) antigen ESAT-6, which has a lower immunogenicity, was able to induce strong antigen-specific Th1 responses, and high titers of antigen-specific IgG1 and IgG2b antibodies, when combined with MPL/DDA [47,48]. In addition, the MPL/DDA formulation has also been reported to activate antigen-specific cytotoxic T-lymphocytes (CTL or activated CD8^+^ T cells) and humoral responses to the malaria sporozoite antigen [41]. However, the virus is too large to encapsulate in liposomes, and it is very difficult to apply to the liposome system because of the irregular aggregation of inactivated viruses during the inactivation process. Although MPL-based liposome adjuvants are a promising system to enhance the cellular immunogenicity of protein- or peptide-based vaccines, we first reported here that this adjuvant system is attractive for enhancing VLP_FMDV_-induced humoral and cellular immune responses. In fact, VLP_FMDV_ produced in *E. coli* has 44.6 nm diameter, which is much smaller than other viral VLPs, such as influenza VLPs (70–200 nm) and human papillomavirus VLPs (55–65 nm) [13,49,50]. Thus, the smaller-sized VLP_FMDV_ is likely to be an important factor in liposome encapsulation.

To date, CD4^+^ and CD8^+^ T cells capable of co-producing IFN-γ, TNF-α and IL-2 (namely multifunctional CD4^+^ and CD8^+^ T cells) have been considered to be important for controlling various viral infections [49,50]. Darrah et al. reported that T cells co-producing IFN-γ, TNF-α and IL-2 cytokines have a greater capacity to aid cognate cells (effector and/or memory cells against pathogens) compared with single- or double-cytokine-producing T cells [28]. In particular, IFN-γ, TNF-α and IL-2 produced by T cells were recently shown to be associated with protection against FMDV infection [51]. For example, inactivated FMDV-vaccinated mice receiving IFN-γ, TNF-α and IL-2 as vaccine adjuvants exhibited a 100% survival rate, whereas inactivated FMDV-only vaccinated mice exhibited a 40% survival rate, which was correlated with FMDV-specific antibody titers, and with levels of memory B cells, memory γδ T cells, and effector/memory T cells. We also demonstrated that the frequency of multifunctional CD4^+^ and CD8^+^ T cells increased in VLP vaccines (MPL/DDA-VLP_FMDV_) formulated with MPL/DDA. Upon stimulation with VLPs, MPL/DDA-VLP_FMDV_-immunized mice displayed significantly greater levels of VLP-specific CD4^+^CD44^+^ and CD8^+^CD44^+^ memory T cells capable of producing single- (in only CD4^+^ T cells), double- (particularly IFN-γ^+^TNF-α^+^ and IFN-γ^+^IL-2^+^ T cells) and triple-positive effector cytokines in the spleen, thereby indicating that the MPL/DDA formulation can be included in interventions capable of promoting multifunctional T cells to control FMDV. Although we addressed much important evidence demonstrating the enhancement of cellular and multifunctional immune responses by the encapsulation of VLP_FMDV_, several additional experiments should be performed to support these results. For example, mice strain-, age-, and sex-dependent differences in MPL/DDA-VLP_FMDV_ vaccine-induced immunity should be investigated to clarify the immunogenicity and vaccine efficacy of MPL/DDA-VLP_FMDV_. In addition, the efficacy of the MPL/DDA-VLP_FMDV_ vaccine against that of a currently inactivated vaccine must be compared by using a challenge animal model, which might explain the advantages of MPL/DDA-VLP_FMDV_ vaccine-induced immunity.

Although the protective roles of Th1 and activated CD8^+^ T cells against FMDV infection have been extensively studied [12,27], relatively little is known concerning the role of the Th17 response. Since IL-17-producing cells are critical for host defense through controlling innate and adaptive immunity against infectious diseases in the mucosa, it might be important when FMDV invades animals through mucosal surfaces [51,52]. For example, Lee et al. showed that inoculation with an inactivated FMDV vaccine and IL-23, which is essential for Th17 cell differentiation, induces strong memory B cell expansion, confers protective immunity, and imparts significant protection upon a murine model against FMDV infection [53]. We found that VLP_FMDV_ alone had a lesser or no impact on Th17 activation, but one formulated with MPL/DDA could induce significant levels of VLP-specific IL-17A-producing CD4^+^ T cells. The protective immune response of VLP against the Asia 1 serotype used in this study was certainly high. Since VLPs have highly strain-specific immunological properties, although an adjuvant effect of MLP/DDA may somehow contribute to the activation of T cells, we should not expect pan-serotype specific effects against multiple FMDV strains other than Asia 1 serotype by MLP/DDA/VLP_FMDV_. However, our present study provided the proof-of-concept for developing an immunologically enhanced and effective FMDV vaccine by employing VLP and MLP plus DDA systems. The subsequent study will be focused on the development of multi-serotype effective FMDV vaccines by including VLPs from multiple prevalent FMDV serotypes.

## 5. Conclusions

Our present data demonstrated that the MPL/DDA formulation shows potential as an effective immunostimulatory adjuvant for VLP_FMDV_ vaccines by satisfying the following criteria: (1) the ability to induce strong antigen-specific Th1 and Th17 immune responses; (2) showing remarkable generation of antigen-specific multifunctional CD4^+^ and CD8^+^ memory T cells; and (3) showing excellent increases in antigen-specific antibody titers. Thus, MPL/DDA might be an excellent adjuvant for VLP_FMDV_ vaccines, especially considering the otherwise low immunogenicity of VLP_FMDV_ vaccine candidates against FMDV.
